# A small intergenic region drives exclusive tissue-specific expression of the adjacent genes in *Arabidopsis thaliana*

**DOI:** 10.1186/1471-2199-10-95

**Published:** 2009-10-16

**Authors:** Hernán G Bondino, Estela M Valle

**Affiliations:** 1 Instituto de Biología Molecular y Celular de Rosario (IBR-CONICET), Facultad de Ciencias Bioquímicas y Farmacéuticas, Universidad Nacional de Rosario, Suipacha 531, S2002LRK Rosario, Argentina

## Abstract

**Background:**

Transcription initiation by RNA polymerase II is unidirectional from most genes. In plants, divergent genes, defined as non-overlapping genes organized head-to-head, are highly represented in the Arabidopsis genome. Nevertheless, there is scarce evidence on functional analyses of these intergenic regions. The At5g06290 and At5g06280 loci are head-to-head oriented and encode a chloroplast-located 2-Cys peroxiredoxin B (2CPB) and a protein of unknown function (PUF), respectively. The 2-Cys peroxiredoxins are proteins involved in redox processes, they are part of the plant antioxidant defence and also act as chaperons. In this study, the transcriptional activity of a small intergenic region (351 bp) shared by *At5g06290 *and *At5g06280 *in *Arabidopsis thaliana *was characterized.

**Results:**

Activity of the intergenic region in both orientations was analyzed by driving the β-glucuronidase (*GUS*) reporter gene during the development and growth of Arabidopsis plants under physiological and stressful conditions. Results have shown that this region drives expression either of *2cpb *or *puf *in photosynthetic or vascular tissues, respectively. *GUS *expression driven by the promoter in *2cpb *orientation was enhanced by heat stress. On the other hand, the promoter in both orientations has shown similar down-regulation of *GUS *expression under low temperatures and other stress conditions such as mannitol, oxidative stress, or fungal elicitor.

**Conclusion:**

The results from this study account for the first evidence of an intergenic region that, in opposite orientation, directs *GUS *expression in different spatially-localized Arabidopsis tissues in a mutually exclusive manner. Additionally, this is the first demonstration of a small intergenic region that drives expression of a gene whose product is involved in the chloroplast antioxidant defence such as *2cpb*. Furthermore, these results contribute to show that *2cpb *is related to the heat stress defensive system in leaves and roots of *Arabidopsis thaliana*.

## Background

A promoter region of an eukaryotic protein-encoding gene usually consists of a core promoter region of around 50 bp nucleotides adjacent to the transcription initiation site, and multiple distal DNA regulatory elements to control transcription efficiency. There are several key genetic elements within a core promoter: the TATA box, an initiator element, the downstream promoter element usually found in TATA-less promoters, and the TFIIB-recognition element [1,2]. The TATA boxes are usually located about 25 to 30 bp upstream of the transcription start site (TSS), while the less conserved initiator elements span the TSS. These sequences contribute to an accurate transcription initiation and to the TATA-containing promoters strength. In Arabidopsis core promoters, the TATA box is located between -50 and -20 relative to the TSS and, instead of the initiator element around the TSS, the YR rule (Y: C or T; R: A or G) applies to most of them. Another element is the pyrimidine patch (Y Patch), although its role is still unknown. These three elements are orientation-sensitive [3]. Other promoter elements found in Arabidopsis and rice are regulatory element groups (REGs), which appear upstream of the TATA box (-20 to -400), and exist in an orientation-insensitive manner [3].

Transcription initiation by RNA polymerase II is unidirectional from most genes. However, several reports indicate that divergent transcription is likely a common feature for active promoters [4-7].

Divergent genes, defined as non-overlapping genes organized head-to-head in opposite orientation, represent a 36.5% of the total gene pairs when separated by less than 1 kb in the Arabidopsis genome [8]. Nevertheless, there is scarce evidence on functional analyses of the intergenic regions between those gene pairs. Previous findings of head-to-head oriented genes sharing an intergenic region with putative bidirectional promoters were reported in *Brassica napus *[9], *Capsicum annuum *[10], and by computational analysis in rice, Arabidopsis, and black cottonwood [11]. Large-scale studies of expression data in Arabidopsis revealed that neighbouring genes in the genome are co-expressed [12], and that the lengths of the intergenic sequences have opposite effects on the ability of a gene to be epigenetically regulated for differential expression [13]. Two recent papers have shown activity of larger intergenic regions in rice (1.8 kbp) and Arabidopsis (2.1 kbp), functioning as bidirectional promoters of chymotrypsin protease inhibitor [14] and chlorophyll a/b-binding protein [15] genes, respectively. These systems were assessed in a heterologous background using onion epidermal cells [14], and also in stable transgenic plants, the latter intended to be used for genetic engineering-based crop improvement [15].

All divergent gene pairs are potential sources of bidirectional promoters. To define the function of the corresponding intergenic regions and their transcriptional regulation is of great interest for plant molecular biologists.

In this study, a divergent promoter of a protein-encoding gene pair (*At5g06290 *and *At5g06280*) with an intergenic region of 351 bp was analyzed. The At5g06290 and At5g06280 loci encode a 2-Cys peroxiredoxin B (2CPB), which are a chloroplast-located protein [16], and a protein of unknown function (PUF), respectively . The 2-Cys peroxiredoxins are proteins involved in redox processes, and their functions are related to the antioxidant defence of the plant [17], photosynthesis, abiotic stress response, and possibly chloroplast-to-cytosol signalling [18]. In yeast, peroxiredoxins could act as molecular chaperons, increasing resistance to heat stress [19]. The expression pattern of the *At5g06290 *and *At5g06280 *was tested by fusing the intergenic region in opposite orientation to β-glucuronidase (*GUS*) reporter gene during the development and growth of Arabidopsis plants as well as during stress situations.

## Results

### Functional analysis of the intergenic region between *At5g06280 *and *At5g06290 *in Arabidopsis plants during their development and growth

**Figure 1:**
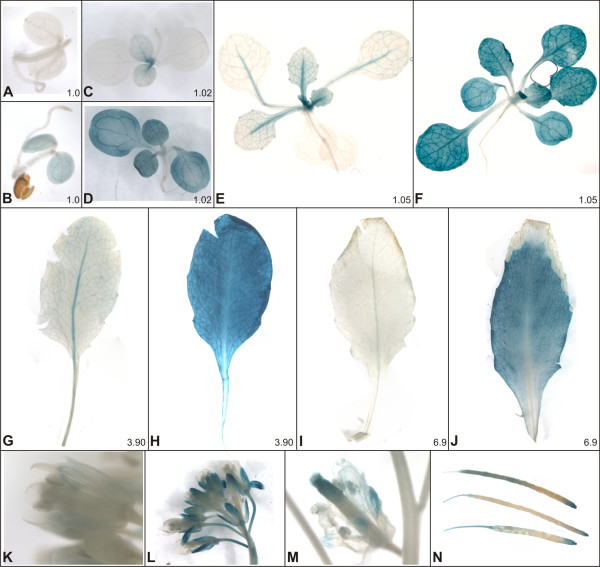
**Expression of *At5g06280 *and *At5g06290 *during life cycle of Arabidopsis**. Histochemical detection of GUS in Arabidopsis plants from *Prom280:GUS *and *Prom290:GUS *lines of different ages. The Arabidopsis growing stages (according to [20]) are indicated at the right bottom corner of the pictures. GUS activity are seen in *Prom280:GUS *line (C, E, G, I) in the petiole and vascular bundle of midrib in all the leaves, but not in the cotyledons (A). The *Prom290:GUS *line (B, D, F, H, J) has evidenced staining in mesophillic tissue of the leaves at all stages. (K) Open flower of *Prom280:GUS *line showing staining of the vascular tissues of the sepals. (L) Open flower of *Prom290:GUS *line with stained sepals. (M) Senescent flower of *Prom290:GUS *line. (N) Siliques of *Prom290:GUS *line showing the stained style and stigmatic tissue. Siliques of *Prom280:GUS *line were not stained at all (data not shown).

To test functionality of the intergenic region shared by the divergent genes *At5g06280 *and *At5g06290 *during Arabidopsis life cycle, the DNA fragment was fused to *GUS *in both orientations (*Prom280:GUS *and *Prom290:GUS*, respectively). Accordingly, we cloned a 530 bp DNA fragment (the 351 bp intergenic region and the 5' untranslated regions) upstream of *GUS *in the binary vector pBI101.1. The constructs were introduced into wild-type Arabidopsis plants by floral dip, multiple transgenic plants were obtained, and more than 3 independent lines were examined for each construct throughout development. GUS staining was performed in Arabidopsis plants during life cycle (Figure 1, stages 1.0 to 6.9 according to [20]). Interestingly, *Prom280:GUS *plants have shown staining almost exclusively in the petiole and vascular bundle of midrib in all the leaves (Figure 1C, 1E, 1G and 1I), sepals (Figure 1K), but not in the cotyledons (Figure 1A), while *Prom290:GUS *plants have shown staining mainly in the leaf mesophyll (Figure 1B, 1D, 1F, 1H and 1J), sepals (Figure 1L), and siliques (Figure 1M and 1N). It is worth noticing that stronger GUS staining was observed for *Prom290:GUS *plants (it was visualized even after three hours of staining) in comparison with *Prom280:GUS *plants at all growth stages (data not shown). Results indicate that the intergenic region between *At5g06290 *and *At5g06280 *directs *GUS *expression in a spatially exclusive manner depending on the promoter orientation during Arabidopsis development and growth (Figure 1).

As 2CPB is a chloroplastic protein [16], we analyzed the putative intracellular location of PUF using ChloroP 1.1 Server [21] and the deduced amino acid sequence of *At5g06280*. The prediction results have shown that PUF (156 residues) is likely to be a plastidic protein, because it has an amino-terminal extension indicative of chloroplast transit peptide (score 0.506). For comparison, 2CPB score was 0.598 using this web tool.

### Response of *Prom280:GUS *and *Prom290:GUS *plants to various stresses

**Figure 2:**
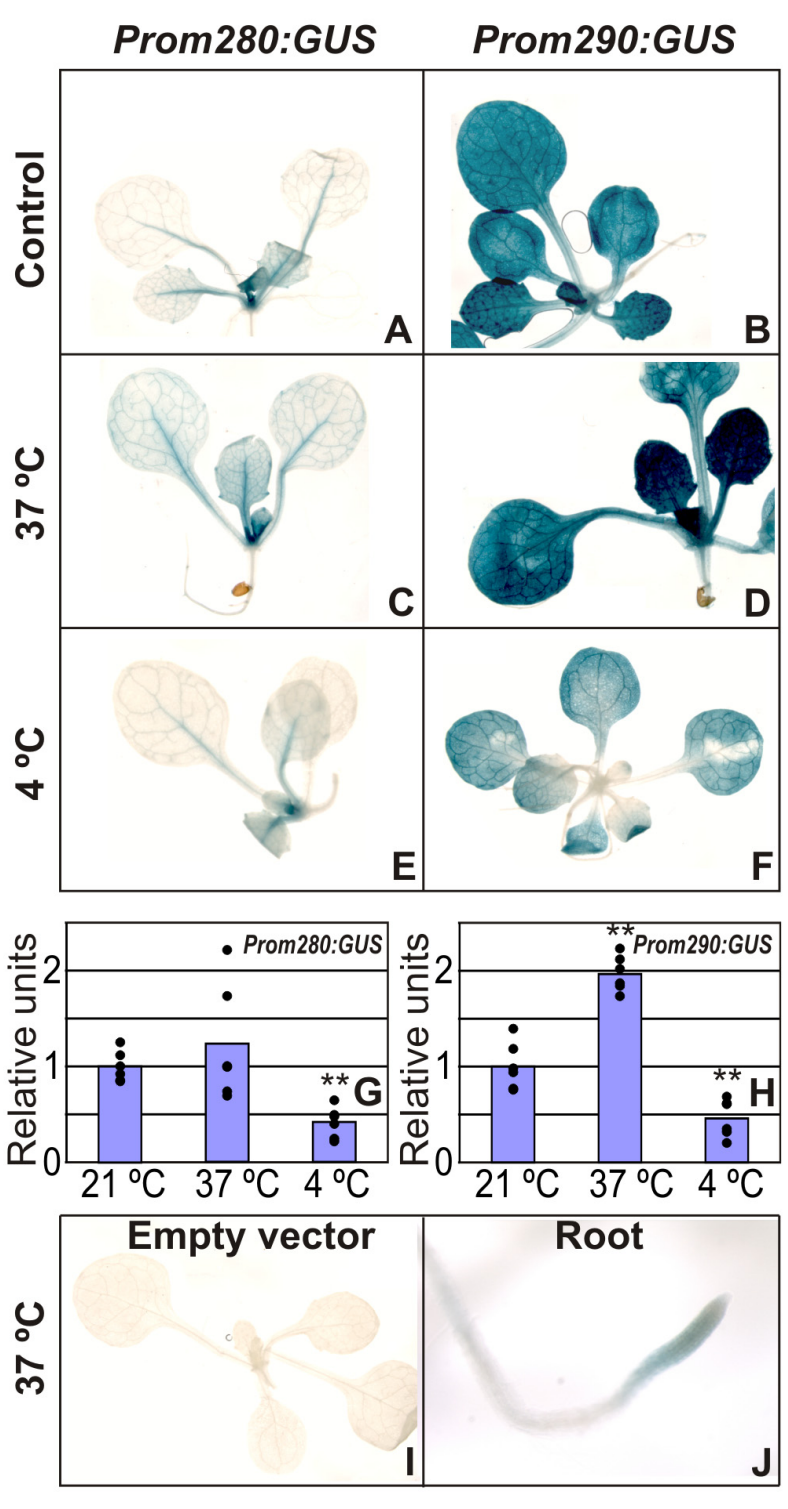
**Expression of *GUS *in *Prom280:GUS *and *Prom290:GUS *plants in response to temperature treatments**. Ten-day-old plants were grown on MS agar plates photoautotrophically at 21°C, and incubated for 48 h at 37°C and 4°C. GUS staining of *Prom280:GUS *and *Prom290:GUS *plants before (A and B) and after transferring the plants to higher (37°C, C and D) or lower (4°C, E and F) temperature conditions. GUS activity was quantified in whole aerial part using 4-methylumbelliferone [MU] as substrate, and results are reported in a relative scale (G and H). Control of *Prom280:GUS *line was 6.52 ± 1.04 nmoles MU/min/mg protein, and control of *Prom290:GUS *line was 76.56 ± 18.84 nmoles MU/min/mg protein. Each point represents a single replicate. Asterisks (**) indicate significant differences between treatments and controls according to Student's t-Test at *P *< 0.005. Plants transformed with pBI101.1 (I). Roots of *Prom290:GUS *line after heat treatment (37°C) (J).

**Figure 3:**
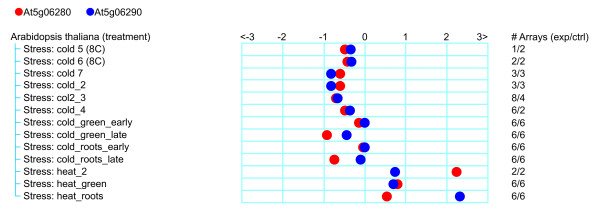
**Expression levels of the genes after heat or cold stress as shown by Genevestigator**. Response viewer of Genevestigator software shows that *At5g06280 *and *At5g06290 *genes decrease their expression levels in all cold stress experiments and increase their levels with heat stress treatments.

Different stress conditions lead to the production of reactive oxygen species (ROS) as a consequence of membrane and protein damage [22]. The expression of 2-Cys peroxiredoxins are reported to be redox regulated [23]. Therefore, it was decided to test the response of *Prom280:GUS *and *Prom290:GUS *plants to various environmental stresses. Firstly, the effect of temperature treatment in 10-day old Arabidopsis seedlings was analyzed. Plants of both transgenic lines were incubated for 48 h at 37°C or 4°C and, after the treatment, they were submitted to GUS staining procedure. Figure 2 shows that leaves from both plant lines were stained stronger under heat stress (Figures 2C and 2D), maintaining the same tissue specificity to the control condition (Figures 2A and 2B). In addition, the root tips were stained in the case of *Prom290:GUS *plants (Figure 2J). In both plant lines the GUS staining pattern was conserved under cold stress (Figures 2E and 2F), although the expression levels were weaker than control conditions as revealed by quantification of the GUS staining intensity (Figures 2G and 2H). Furthermore, no expression was detected in the plants carrying the vector without the intergenic region (empty vector) (Figure 2I). Further analysis of *puf *and *2cpb *expression using the response viewer of Genevestigator software [24] is presented in Figure 3. Under several cold treatments, the aerial part of Arabidopsis plants have evidenced decreased expression of *puf *and *2cpb*, while under heat conditions, the plants have evidenced enhanced expression of both genes (Figure 3). Similar responses were observed in the expression of *GUS *in *Prom280:GUS *and *Prom290:GUS *plants submitted to temperature stress (Figure 2). Additionally, in roots of *Prom290:GUS *plants, the expression of *2cpb *is markedly increased by heat stress (Figure 2J), which is consistent with data obtained from roots under the same stress treatment (Figure 3).

To confirm the effect of heat treatment on the induction of 2CPB, 10-day old wild-type Arabidopsis plants were submitted for 2 days at 37°C, and the total protein of leaves and roots were extracted and analyzed by SDS-PAGE and immunoblotting. Results are presented in Additional file 1. The total protein pattern has shown slight differences between control and treated plants in the leaf or root tissues, especially in higher molecular masses larger than 66 kDa. Immunoblot analysis of these tissues has shown induction of 2CPB in both leaves and roots after heat treatment (Additional file 1, bottom panel). These data indicate that heat treatment was able to increase not only 2CPB protein level in root and leaf of wild-type plants (Additional file 1), but also GUS activity in the same tissues as observed in *Prom290:GUS *plants (Figures 2D and 2J).

**Figure 4:**
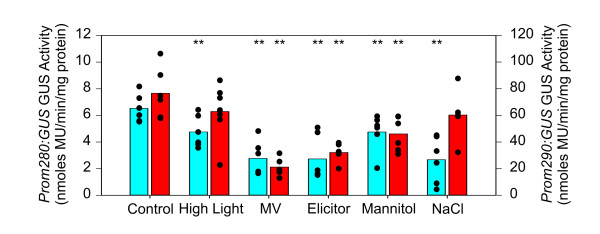
**Effect of several stresses on *GUS *expression in *Prom280:GUS *and *Prom290:GUS *lines**. Ten-day-old Arabidopsis seedlings were grown on MS agar at 21°C and incubated for 6 h to high light (800 μmol m^-2 ^s^-1^). For other stress conditions, Arabidopsis plants were cultivated on MS agar supplemented with 50 mM NaCl, 0.1 μM MV, 100 mM mannitol or 1.3 mg/mL elicitors. GUS activity was quantified in whole aerial part using MU as substrate. Each point on the bar represents a single replicate (blue bars for *Prom280:GUS *line and red bars for *Prom290:GUS *line). Asterisks (**) indicate significant differences between treatments and controls according to Student's t-Test at *P *< 0.05. Note the scale difference between *Prom280:GUS *and *Prom290:GUS *lines.

Other sources of ROS are biotic and abiotic stresses. The effect of different stress conditions on the expression levels of *Prom280:GUS *and *Prom290:GUS *plants were evaluated, and the results are presented in Figure 4. *GUS *expression was similarly reduced in both Arabidopsis lines under oxidative stress caused by methyl viologen (MV), a redox cycling compound, fungal elicitor, and mannitol. Additionally, GUS expression was down-regulated by high light and NaCl in *Prom280:GUS *lines, while in *Prom290:GUS *lines were unaffected. It is worth mentioning that the expression of *GUS *in *Prom280:GUS *lines was ten times lower than in *Prom290:GUS *plants when calculated per mg of protein of the aerial parts of the plant. This could be due to a dilution effect of GUS activity specifically located in vascular bundles of the leaf in *Prom280:GUS *lines, in comparison with the whole leaf expression pattern of GUS in *Prom290:GUS *plants.

These results suggest that *puf *and *2cpb *are stress-responsive genes, although they are not always affected in the same way by the same stress conditions.

### In search of *cis*-elements in the promoter of *puf *and *2cpb*

**Figure 5:**
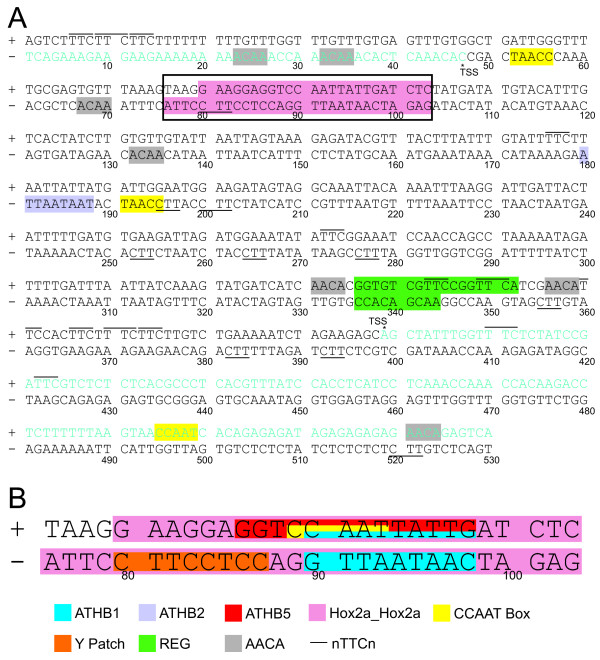
**Intergenic sequence and 5'UTRs**. The double strand sequence of the intergenic and the 5'UTR regions of *2cpb *and *puf *are shown in A. The *cis*-elements found in the analyzed region are indicated at the bottom of the figure. More details of the 28 bp region (enclosed) are shown in B. The plus (+) strand is upstream of *At5g06290 *and the minus strand is upstream of *At5g06280*. The 5'UTRs are shown in light green. Asterisks indicate the TSS. No TATA boxes have been found. ATHB1 is the binding site of the transcription factor ATHB1, which is involved in differentiation of the palisade mesophyll cells and leaf development. ATHB2 is the binding site of the transcription factor ATHB2, which is an element of response to far-red light. ATHB5 is the binding site of the transcription factor ATHB5, which is involved in the regulation of light-dependent developmental phenomena. Hox2a_Hox2a is the binding site of proteins with the homeodomains ZmHOX2a(1) and ZmHOX2a(2). CCAAT box is found in the promoter of *Hsp *genes. Y Patch is a direction-sensitive plant core promoter element that appears around TSS. REG is a direction-insensitive element that is preferentially found around -20 to -400 bp relative to TSS. AACA is a negative regulatory element in vascular promoters that repress activity in other cell types. The yeast heat shock factor 1 binding sequence nTTCn is underlined in the minus strand and overlined in the plus strand.

*In silico *analysis of the divergent promoter was performed looking for *cis*-elements using the Plant Promoter Database (ppdb) [25], PlantCARE [26], PLACE [27], and Athamap [28] web tools. Analysis revealed no TATA box available. The elements distribution in the 530 bp region is shown in Figure 5. We identified binding sites for four homeodomain-leucine zipper transcription factors: ATHB1, which was reported to be involved in differentiation of the palisade mesophyll cells and leaf development [29,30]; ATHB2, which is responsive to far-red light [31]; ATHB5, which is a transcription factor involved in the regulation of light-dependent developmental phenomena [29]; and transcription factors similar to ZmHox2a, which have the homeodomains ZmHOX2a(1) and ZmHOX2a(2) [32]. Furthermore, a Y Patch near *puf *TSS, and seven REGs near *2cpb *TSS were identified; however, their functions are still unknown. An AACA element, which was described as a negative regulatory element in vascular promoters that represses activity in other cell types [33], were identified in seven positions. Lastly, a CCAAT box, present in the promoter of heat shock protein (Hsp) genes [34], was found four times, and the nCTTn element present in the promoters of several *Hsp *genes [35] was found 23 times. This analysis displayed no other overrepresented *cis*-element in the promoter region under study.

### Distribution of distances between genes and their nearest neighbours in Arabidopsis genome

**Figure 6:**
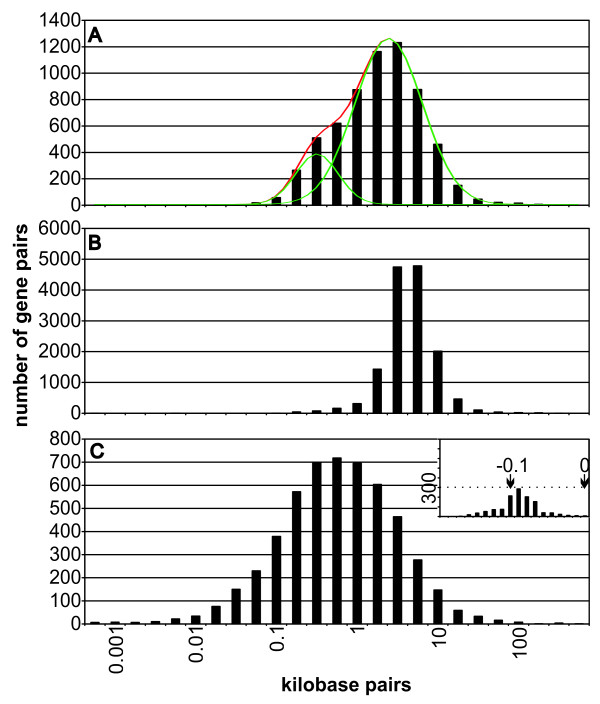
**Distribution of distances between genes and their nearest neighbours in Arabidopsis**. (A) The distribution of distances between 5'ends of genes on opposite strands is deconvoluted in two peaks showing a peak centred at 323 bp and another at 2,518 bp, the 38.4% of the genes pairs are closer than 1,000 bp. (B) Distances between 5'ends of genes on the same strand showing that 4.3% of gene pairs are closer than 1,000 bp. (C) Analysis of the distribution of distances between 3'ends of genes on opposite strands showing that 75% of the gene pairs are closer than 1,000 bp. Inset: indicate the distribution of the overlapping genes.

To further characterize this 351 bp promoter on genome-wide scale, the distribution of intergenic regions of similar lengths into the Arabidopsis genome was studied. For that purpose, the distribution of distances between Arabidopsis genes and their nearest neighbours in the same and opposite strands were explored. The distances between the TSS of the nearest gene neighbours for each of the 27,141 genes predicted (see Methods) after filtering out genes annotated as pseudogenes and transposons were calculated. The distribution of distances between 5'ends of genes on opposite strands is bimodal, which could be deconvoluted in two peaks centred at 323 bp (around 140 gene pairs between 300 and 350 bp length) and 2.5 kbp (Figure 6A). This type of distribution was not present in all the around 14,000 genes with the nearest neighbours on the same strand (Figure 6B), or when the distances were calculated between the 3'ends of the genes on opposite strands (Figure 6C). Noticeably, only 4.3% of the gene pairs with 5'ends on the same strand are closer than 1,000 bp (Figure 6B), while 75% of the gene pairs with 3'ends on opposite strands are closer than 1,000 bp, with 1,234 of them having overlapping regions (Figure 6C, inset). We designated the region between the two non-overlapping 5'ends of genes located on opposite strands as a putative bidirectional promoter. This analysis shows that out of 6,438 divergent gene pairs (Figure 6A), 2,469 are putative bidirectional promoters of less than 1,000 bp in the Arabidopsis genome. Most of the head-to-head oriented genes (98%) have predictably shown non-overlapping bidirectional promoters, and only 874 (13.8%) gene pairs are less than 323 bp in length.

## Discussion

With the availability of complete genome sequences for a number of organisms, functionality of intergenic regions has attracted more attention. Computational analysis has shown that divergent gene pairs with intergenic regions less than 1 kb are quite abundant in the sequenced eukaryotic genomes of both plants and animals [5,8]. The interest in studying intergenic region functionality is increasing not only to better understand divergent transcription, but also to use them as a new toolkit to manipulate genomes [36]. In plants, particularly, very few reports about this matter are available. An example of such investigations in plants in which data from computational assistance and bidirectionalization were integrated to construct a synthetic transcriptional unit for high-level reporter-gene expression in response to specific elicitors was reported, thus yielding exciting results [37]. In this study, it has been found that the region shared by two divergent genes in the chromosome 5 of *Arabidopsis thaliana *(*At5g06280 *and *At5g06290*) functions as a promoter in both orientations. In addition, this study was able to demonstrate that tissue and developmental expression patterns differed between *puf *and *2cpb*. Head-to-head genes from other organisms such as human, mouse, and rat genomes statistically tend to perform similar functions, and gene pairs associated with the significant co-functions seem to have stronger expression correlations [38]. In this case, the gene products of *At5g06280 *and *At5g06290 *are both presumably located in the chloroplasts, although it is unknown if their functions are related. Thus, it is known that 2CPB is located in the chloroplasts and prevents oxidative damage of chloroplast proteins [17]. The transcript increase of *2cpb *was correlated with chlorophyll distribution and also accumulated in plants with decreased catalase activity and upon heat stress [39]. Down-regulation of *2cpb *was observed upon pathogen infection, ozone and cold [40,41]. Instead, the role of PUF remains unknown until today, and presumably it would be a chloroplast-located protein as predicted by ChloroP analysis [21].

When searching for *At5g06280 *and *At5g06290 *potential orthologues, it has been found that this head-to-head gene organization was not conserved among other genomes (data not shown); pointing out that most probably their gene products are not functionally related. In humans, analysis of genome-wide expression data demonstrated that a minority of bidirectional gene pairs are expressed through a mutually exclusive mechanism [5]. In this study, the tissue-specific expression of both genes directed by the divergent promoter has shown unidirectional activity for *puf *in petiole and vascular bundles and unidirectional activity in the opposite direction in different tissues for *2cpb*. The higher expression of *2cpb *in the leaf mesophyll, but not in vascular bundles, is coincident with its function in the redox processes of chloroplasts [40]. Taken together, these results suggest that the directionality of the promoter activity may be regulated to some degree in a tissue-specific manner. In fact, a *cis*-motif associated to vascular bundle expression (AACA) [33] was found several times in the *puf *direction of transcription.

Furthermore, it has been demonstrated that the divergent promoter shared by *puf *and *2cpb *responded to temperature stress. In relation to this, the higher 2CPB levels in the leaf and root caused by heat treatment of Arabidopsis seedlings would indicate a role of this protein in temperature stress. In yeast, peroxiredoxins could alternatively function as peroxidases and molecular chaperons, increasing resistance to heat stress [19]. It is well known that exposure of plants to high temperature leads to the production of Hsps. The yeast heat shock factor 1 binding sequence nTTCn (or nGAAn) [35] was found highly represented in the intergenic region of this study. Therefore, it is tempting to speculate that high temperature could stimulate *2cpb *similarly to *Hsp *genes. Remarkably, the *puf *expression was repressed similarly to *2cpb *by several stress conditions.

*In silico *analysis of this promoter using ppdb revealed that it is a TATA-less promoter in both orientations. In plant genomes putative bidirectional promoters have TATA boxes underrepresented [11]. A recent study [42] suggested that TATA box-containing genes have longer intergenic upstream regions and increased variation across species because their upstream regulatory potential is greater and, therefore, more amenable to change and modulation. The TATA box appears to be responsible for promoter unidirectionality in most cases, whereas having no TATA boxes appears to be a novel mechanism of regulation by bidirectional promoters compared to unidirectional promoters. This analysis also revealed that in a short region of this promoter (28 bp) (Figure 5B), four different *cis*-elements are overlapped. They are: one heat shock element (CCAAT box), a Y Patch found in the majority of Arabidopsis promoters but with unknown function [25], and three binding sites of homeodomains-leucine zipper transcription factors, some of them being able to bind in both directions [27,28]. These *cis*-elements would be leading the transcription of *2cpb*, specially ATHB1, which is involved in differentiation of the palisade mesophyll cells, and ATHB5, which in turn is involved in the control of leaf morphology development [26]. Upstream of this region there are three AACA elements in the +/- 25 bp region of *puf *TSS (Figure 5A). This is a negative regulatory element in vascular promoters, which represses activity in other cell types [33] suggesting that, in the intergenic region under analysis, this *cis*-element would be preventing *puf *transcription in mesophilic cells. The expression of *puf *in vascular bundle of midribs could be activated by ATHB2, which has a homeodomain too, and by the Y Patch that is located in the 28 bp region above mentioned. The *2cpb *and *puf *putative promoter regions mentioned have an element of response to heat near them, which could explain the heat stress experiments. It was not possible to find any abiotic stress element overrepresented in the 530 bp region analyzed, suggesting that the expression pattern observed in Figure 4 could be the result of the complex interaction of the transcription factors that bind the 28 bp region. Overall, results obtained from this study indicate that the multiple stress responsiveness of the intergenic region would reside within the 351 bp.

When length is considered, the short promoter shared by *2cpb *and *puf *belongs to a minority group of putative bidirectional promoters present in the Arabidopsis genomes. In fact, Arabidopsis genome has a bimodal distribution of distances between the 5'ends of genes on opposite strands, peaking the smaller group of gene pairs at 323 bp. This is the first intergenic region functionally studied of this small group of Arabidopsis promoters. Plants are sessile organisms and, during their growth, they occasionally are affected by adverse environmental conditions; therefore, they may rely more strongly on elaborate transcriptional response programs to survive. Then, it is highly possible that other intergenic regions of similar lengths and regulatory features could be found in plants.

## Conclusion

In this report, it has been shown that a 351 bp intergenic region between head-to-head oriented *At5g06290 *and *At5g06280 *directs genes expression in different Arabidopsis tissues in a mutually exclusive manner. Gene products of these loci are a chloroplast-located 2-Cys peroxiredoxin B involved in the antioxidant defence, and a protein of unknown function. This is the first report of an intergenic region that drives expression of a gene involved in the chloroplast antioxidant defence. These results also show that 2CPB is induced by heat stress in the leaves and roots, suggesting a function for this protein in the heat stress defensive system of *Arabidopsis thaliana*.

## Methods

### Plant material and growth conditions

*Arabidopsis thaliana *ecotype Columbia (Col-7) was synchronously germinated at 4°C for 48 h and grown in soil-vermiculite mixture (2:1 v/v) in growth chambers at 20-22°C, under long day conditions (16 h light/8 h darkness). The light intensity was set at 130 μmol m^-2 ^s^-1^.

When assaying stress treatments, Arabidopsis plants grown photoautotrophically on agar medium containing 0.5 X Murashige and Skoog (MS) salts (Sigma-Aldrich).

### Stress treatments

Arabidopsis plants were cultivated on agar supplemented with the stress agent: osmotic stress (100 mM mannitol), salt stress (50 mM NaCl), oxidative stress (0.1 μM methyl viologen) or fungal elicitor (1.3 mg/mL autoclaved cellulase, Onozuka R-10, Yakult Honsha, Tokio, Japan). For cold (4°C) and high (37°C) temperature stresses, the plants were grown for 10 days on MS agar without supplements under control conditions and then the temperature treatment was applied for 2 days. For higher light intensity (800 μmol m^-2 ^s^-1^), the plants were grown for 10 days and the treatment was applied for 6 h.

### DNA constructs

The intergenic region with the 5'UTR regions of the genes *At5g06280 *and *At5g06290 *was isolated by PCR from an *A. thaliana *DNA CTAB preparation [43] using the primers 5'-CGC**GGATCC**AGTCTTTCTTCTTCTTTTTTTTTG-3' and 5'-CGC**GGATCC**TGACTCTGTTCTCTCTCTCTATC-3' (added *Bam*HI restriction site in bold). The PCR product was subcloned into pGEM-T Easy Vector (Promega, Madison, USA). DNA sequencing was used to confirm that no spurious mutations were introduced during amplification. The fragment was excised with *Bam*HI, and the 530 bp fragments were cloned into the *Bam*HI site of pBI101.1 to create the plasmids pBI280 and pBI290. The orientation of the fragment was analyzed by PCR with primers that hybridize in the pBI101.1 plasmid (5'-ACAGTTTTCGCGATCCAGAC-3' and 5'-TTATGCTTCCGGCTCGTATG-3') and the primers previously described. *Escherichia coli *strain DH5α was used for plasmid construction. *Agrobacterium tumefaciens *strain GV3101 pMP90 was transformed with plasmids by electroporation, and Arabidopsis (Col-7) plants were transformed by floral dip infiltration [44] with the plasmids pBI101.1, pBI280, or pBI290.

### Histochemical localization of GUS activity

GUS activity was localized by staining the tissues with 0.5 mg of 5-bromo-4-chloro-3-indolyl-b-D-glucuronic acid (X-Gluc; Gold Biotechnology, St Louis, MO, USA) per mL in X-Gluc buffer containing 50 mM sodium phosphate (pH 7.2), 10 mM EDTA, 0.33 mg/mL potassium ferricyanide and 0.001% Tween 20. The tissues were vacuum-infiltrated for three rounds of one min each, and staining reactions proceeded overnight at 37°C. Chlorophyll was removed by soaking in ethanol. The photographs were taken with a binocular microscope Leika MZ16F.

### Analysis of GUS activity

Quantitative analysis of GUS activity was performed on whole aerial part using the GUS activity assay [45], the experiment was made twice, each treatment had three biological replicates and each replicate was a pool of 10 Arabidopsis plants, except the high light treatment which had four biological replicates.

Production of 4-methylumbelliferone [MU] was measured using a DTX 880 Multimode Detector (Beckman Coulter, Fullerton, CA). Protein concentrations of the samples were determined using Bradford reagent [46] and BSA as a standard. The amount of MU was determined from a standard curve, and GUS activity was expressed as nmol MU/min/mg protein. The empty vector transformed plants shown a basal activity of 0.22 ± 0.08 nmoles MU/min/mg protein.

### Immunoblot analysis

To measure the protein levels of 2CPB, 100 mg of tissue were ground to a fine powder in liquid N_2 _and then homogenized with 0.2 mL of buffer (25 mM Hepes (pH 7.5), 0.6 M mannitol, 0.462 mg/mL dithiothreitol, 2 mM EDTA, 0.175 mg/mL phenylmethylsulphonyl fluoride and 1% (w/v) polyvinylpolypyrrolidone). The homogenates were centrifuged at 15,000 *g *for 20 min, and the supernatant protein concentration was determined utilizing BSA as a standard protein as described by [46]. The supernatant was mixed with sample buffer 10× (250 mM Tris-HCl (pH 6.8), 10% SDS, 0.5% bromophenol blue and 20% glycerol), boiled for 5 min, and separated in a 12% SDS-PAGE as described earlier [47]. The gels were stained with Coomassie Brilliant Blue R-250. For immunoblotting, the proteins were transferred to nitrocellulose membranes using a Mini Trans-Blot cell (Bio-Rad, CA, USA) at 100 mA for 100 min. The membranes were treated with polyclonal antibody raised against rapeseed 2-Cys peroxiredoxin [48]. Signals on the membranes were visualized with alkaline phosphatase-conjugated goat anti-rabbit IgG (SIGMA, St Louis, MO, USA).

The signal intensities were quantified from the immunoblot using the Gel-Pro Analyzer software (Media Cybernetics Inc, Silver Spring, MD) and normalized to the intensities observed in control conditions. A representative example from three independent experiments is shown.

### Promoter sequence analysis

The promoter sequence was analyzed using publicly available databases, PlantCARE [26] and PLACE [27], which are databases of plant *cis*-acting regulatory elements; AthaMap [28], which provides a genome-wide map of potential transcription factor binding sites in *Arabidopsis thaliana*; and Plant Promoter Database (ppdb) [25], which is based on species-specific sets of promoter elements, rather than on general motifs for multiple species.

### Arabidopsis promoters length analysis

Annotation data for the *Arabidopsis thaliana *genes was downloaded from The Arabidopsis Information Resource (TAIR) FTP server . The analysis was performed on 27,141 genes after filtering out pseudogenes and transposon-related genes  from 31,762 annotated genes. Start and stop positions of the transcription units along with information on the strand that encodes an mRNA were extracted. Microsoft Office Excel was used to calculate the distances between the 3' ends of the nearest neighbour genes and the distances between 5' ends of the neighbour genes. The overlapping genes were analyzed only in the graph corresponding to the 3'ends of the nearest neighbour genes and the resulting distances among them were less than zero (shown in Figure 6C, inset).

## Authors' contributions

HGB has made substantial contributions to the conception and design of experiments, acquisition of data, analysis and interpretation of data, as well as drafting the manuscript; EMV has been involved in the design of the experiments, analysis and interpretation of data, as well as drafting the manuscript. Both authors have approved the final manuscript.

## Supplementary Material

Additional file 1 **Additional Figure - Immunoblot analysis of 2-Cys peroxiredoxin abundance in leaf and root under heat stress**. Total protein extracts of leaf and root of wild-type Arabidopsis plants were submitted to SDS-PAGE and immunoblot analysis using polyclonal antibodies against rapeseed 2-Cys peroxiredoxin. Line 1, control leaf. Line 2, heat stress treated leaf. Line 3, control root. Line 4, heat stress treated root. The 2-Cys peroxiredoxin levels in root and leaf were higher after heat treatment (lines 2 and 4), the numbers give the means of the percentage of signal intensity relative to each control (lines 1 and 3, respectively) in three independent experiments. Click here for file

## References

[B1] Novina CD, Roy AL (1996). Core promoters and transcriptional control. Trends Genet.

[B2] Smale ST, Kadonaga JT (2003). The RNA polymerase II core promoter. Annu Rev Biochem.

[B3] Yamamoto YY, Ichida H, Matsui M, Obokata J, Sakurai T, Satou M, Seki M, Shinozaki K, Abe T (2007). Identification of plant promoter constituents by analysis of local distribution of short sequences. BMC Genomics.

[B4] Beck CF, Warren RA (1988). Divergent promoters, a common form of gene organization. Microbiol Rev.

[B5] Trinklein ND, Aldred SF, Hartman SJ, Schroeder DI, Otillar RP, Myers RM (2004). An abundance of bidirectional promoters in the human genome. Genome Res.

[B6] Core LJ, Waterfall JJ, Lis JT (2008). Nascent RNA sequencing reveals widespread pausing and divergent initiation at human promoters. Science.

[B7] Seila AC, Core LJ, Lis JT, Sharp PA (2009). Divergent transcription: a new feature of active promoters. Cell Cycle.

[B8] Krom N, Ramakrishna W (2008). Comparative analysis of divergent and convergent gene pairs and their expression patterns in rice, Arabidopsis, and populus. Plant Physiol.

[B9] Keddie JS, Tsiantis M, Piffanelli P, Cella R, Hatzopoulos P, Murphy DJ (1994). A seed-specific Brassica napus oleosin promoter interacts with a G-box-specific protein and may be bi-directional. Plant Mol Biol.

[B10] Shin R, Kim MJ, Paek KH (2003). The CaTin1 (Capsicum annuum TMV-induced Clone 1) and CaTin1-2 Genes are linked head-to-head and share a bidirectional promoter. Plant Cell Physiol.

[B11] Dhadi SR, Krom N, Ramakrishna W (2009). Genome-wide comparative analysis of putative bidirectional promoters from rice, Arabidopsis and Populus. Gene.

[B12] Williams EJ, Bowles DJ (2004). Coexpression of neighboring genes in the genome of Arabidopsis thaliana. Genome Res.

[B13] Colinas J, Schmidler SC, Bohrer G, Iordanov B, Benfey PN (2008). Intergenic and genic sequence lengths have opposite relationships with respect to gene expression. PLoS ONE.

[B14] Singh A, Sahi C, Grover A (2009). Chymotrypsin protease inhibitor gene family in rice: Genomic organization and evidence for the presence of a bidirectional promoter shared between two chymotrypsin protease inhibitor genes. Gene.

[B15] Mitra A, Han J, Zhang ZJ, Mitra A (2009). The intergenic region of Arabidopsis thaliana cab1 and cab2 divergent genes functions as a bidirectional promoter. Planta.

[B16] Dietz KJ, Horling F, Konig J, Baier M (2002). The function of the chloroplast 2-cysteine peroxiredoxin in peroxide detoxification and its regulation. J Exp Bot.

[B17] Baier M, Dietz KJ (1999). Protective function of chloroplast 2-cysteine peroxiredoxin in photosynthesis. Evidence from transgenic Arabidopsis. Plant Physiol.

[B18] Dietz KJ (2007). The dual function of plant peroxiredoxins in antioxidant defence and redox signaling. Subcell Biochem.

[B19] Jang HH, Lee KO, Chi YH, Jung BG, Park SK, Park JH, Lee JR, Lee SS, Moon JC, Yun JW, Choi YO, Kim WY, Kang JS, Cheong GW, Yun DJ, Rhee SG, Cho MJ, Lee SY (2004). Two enzymes in one; two yeast peroxiredoxins display oxidative stress-dependent switching from a peroxidase to a molecular chaperone function. Cell.

[B20] Boyes DC, Zayed AM, Ascenzi R, McCaskill AJ, Hoffman NE, Davis KR, Gorlach J (2001). Growth stage-based phenotypic analysis of Arabidopsis: a model for high throughput functional genomics in plants. Plant Cell.

[B21] Emanuelsson O, Nielsen H, von HG (1999). ChloroP, a neural network-based method for predicting chloroplast transit peptides and their cleavage sites. Protein Sci.

[B22] Apel K, Hirt H (2004). Reactive oxygen species: metabolism, oxidative stress, and signal transduction. Annu Rev Plant Biol.

[B23] Horling F, Baier M, Dietz KJ (2001). Redox-regulation of the expression of the peroxide-detoxifying chloroplast 2-cys peroxiredoxin in the liverwort Riccia fluitans. Planta.

[B24] Zimmermann P, Hennig L, Gruissem W (2005). Gene-expression analysis and network discovery using Genevestigator. Trends Plant Sci.

[B25] Yamamoto YY, Obokata J (2008). ppdb: a plant promoter database. Nucleic Acids Res.

[B26] Rombauts S, Florquin K, Lescot M, Marchal K, Rouze P, Peer Y Van de (2003). Computational Approaches to Identify Promoters and *cis*-Regulatory Elements in Plant Genomes. Plant Physiology.

[B27] Higo K, Ugawa Y, Iwamoto M, Korenaga T (1999). Plant *cis*-acting regulatory DNA elements (PLACE) database: 1999. Nucl Acids Res.

[B28] Steffens NO, Galuschka C, Schindler M, Bulow L, Hehl R (2004). AthaMap: an online resource for in silico transcription factor binding sites in the *Arabidopsis thaliana *genome. Nucleic Acids Res.

[B29] Henriksson E, Olsson AS, Johannesson H, Johansson H, Hanson J, Engstrom P, Soderman E (2005). Homeodomain leucine zipper class I genes in Arabidopsis. Expression patterns and phylogenetic relationships. Plant Physiol.

[B30] Aoyama T, Dong CH, Wu Y, Carabelli M, Sessa G, Ruberti I, Morelli G, Chua NH (1995). Ectopic expression of the Arabidopsis transcriptional activator Athb-1 alters leaf cell fate in tobacco. Plant Cell.

[B31] Carabelli M, Morelli G, Whitelam G, Ruberti I (1996). Twilight-zone and canopy shade induction of the Athb-2 homeobox gene in green plants. Proc Natl Acad Sci USA.

[B32] Kirch T, Bitter S, Kisters-Woike B, Werr W (1998). The two homeodomains of the ZmHox2a gene from maize originated as an internal gene duplication and have evolved different target site specificities. Nucleic Acids Res.

[B33] Scarpella E, Simons EJ, Meijer AH (2005). Multiple regulatory elements contribute to the vascular-specific expression of the rice HD-Zip gene Oshox1 in Arabidopsis. Plant Cell Physiol.

[B34] Rieping M, Schoffl F (1992). Synergistic effect of upstream sequences, CCAAT box elements, and HSE sequences for enhanced expression of chimaeric heat shock genes in transgenic tobacco. Mol Gen Genet.

[B35] Yamamoto A, Mizukami Y, Sakurai H (2005). Identification of a novel class of target genes and a novel type of binding sequence of heat shock transcription factor in *Saccharomyces cerevisiae*. J Biol Chem.

[B36] Venter M (2007). Synthetic promoters: genetic control through *cis *engineering. Trends Plant Sci.

[B37] Chaturvedi CP, Sawant SV, Kiran K, Mehrotra R, Lodhi N, Ansari SA, Tuli R (2006). Analysis of polarity in the expression from a multifactorial bidirectional promoter designed for high-level expression of transgenes in plants. J Biotechnol.

[B38] Li YY, Yu H, Guo ZM, Guo TQ, Tu K, Li YX (2006). Systematic analysis of head-to-head gene organization: evolutionary conservation and potential biological relevance. PLoS Comput Biol.

[B39] Mittler R, Vanderauwera S, Gollery M, Van BF (2004). Reactive oxygen gene network of plants. Trends Plant Sci.

[B40] Dietz KJ, Jacob S, Oelze ML, Laxa M, Tognetti V, de Miranda SM, Baier M, Finkemeier I (2006). The function of peroxiredoxins in plant organelle redox metabolism. J Exp Bot.

[B41] Goulas E, Schubert M, Kieselbach T, Kleczkowski LA, Gardestrom P, Schroder W, Hurry V (2006). The chloroplast lumen and stromal proteomes of *Arabidopsis thaliana *show differential sensitivity to short- and long-term exposure to low temperature. Plant J.

[B42] Walther D, Brunnemann R, Selbig J (2007). The regulatory code for transcriptional response diversity and its relation to genome structural properties in *A. thaliana*. PLoS Genet.

[B43] Stewart CN, Via LE (1993). A rapid CTAB DNA isolation technique useful for RAPD fingerprinting and other PCR applications. Biotechniques.

[B44] Clough SJ, Bent AF (1998). Floral dip: a simplified method for Agrobacterium-mediated transformation of *Arabidopsis thaliana*. Plant J.

[B45] Weigel D, Glazebrook J (2002). Arabidopsis: a laboratory manual.

[B46] Bradford MM (1976). A rapid and sensitive method for the quantitation of microgram quantities of protein utilizing the principle of protein-dye binding. Anal Biochem.

[B47] Laemmli UK (1970). Cleavage of Structural Proteins during the Assembly of the Head of Bacteriophage T4. Nature.

[B48] Caporaletti D, D'Alessio AC, Rodriguez-Suarez RJ, Senn AM, Duek PD, Wolosiuk RA (2007). Non-reductive modulation of chloroplast fructose-1,6-bisphosphatase by 2-Cys peroxiredoxin. Biochem Biophys Res Commun.

