# Supplementary figures and images for: A small intergenic region drives exclusive tissue-specific expression of the adjacent genes in Arabidopsis thaliana
Source: BMC Mol Biol. 2009 Oct 16;10:95. doi: 10.1186/1471-2199-10-95 (PMC2772851; doi:10.1186/1471-2199-10-95)

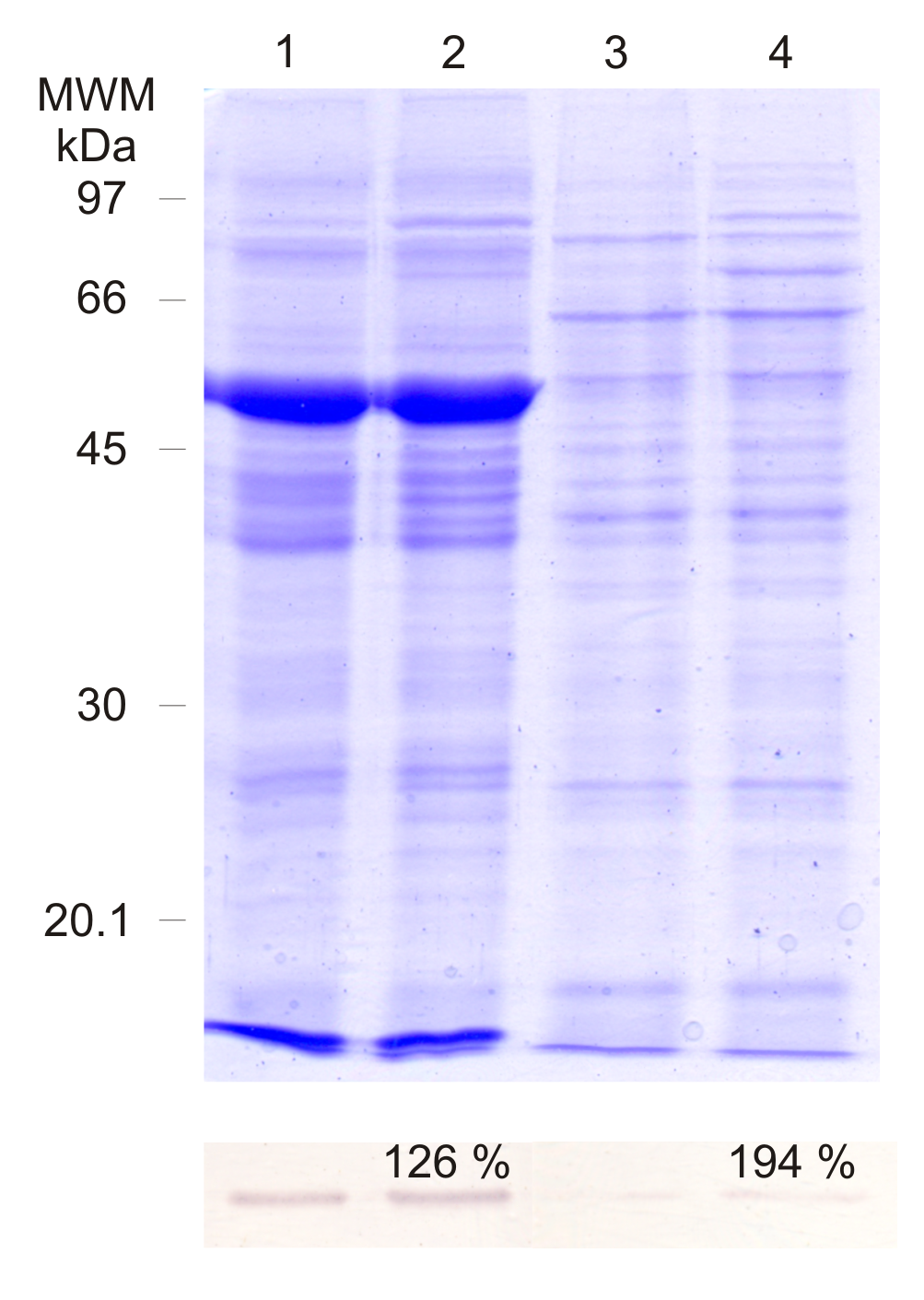

Supplement: Additional file 1 — Additional Figure - Immunoblot analysis of 2-Cys peroxiredoxin abundance in leaf and root under heat stress. Total protein extracts of leaf and root of wild-type Arabidopsis plants were submitted to SDS-PAGE and immunoblot analysis using polyclonal antibodies against rapeseed 2-Cys peroxiredoxin. Line 1, control leaf. Line 2, heat stress treated leaf. Line 3, control root. Line 4, heat stress treated root. The 2-Cys peroxiredoxin levels in root and leaf were higher after heat treatment (lines 2 and 4), the numbers give the means of the percentage of signal intensity relative to each control (lines 1 and 3, respectively) in three independent experiments. [file 1471-2199-10-95-S1.TIFF]
